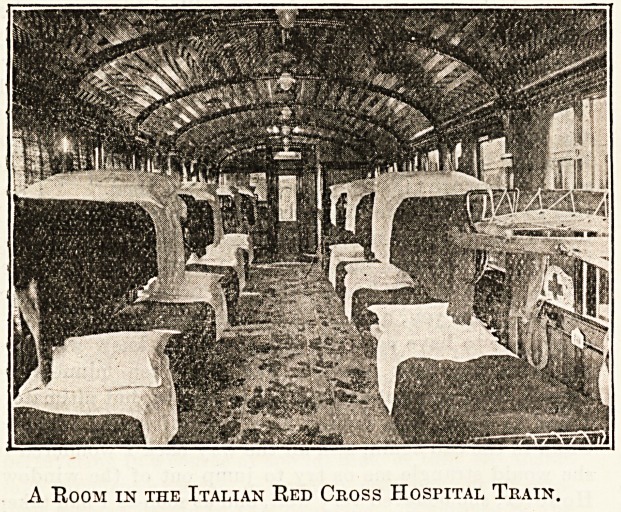# "The Hospital" Nursing Section

**Published:** 1906-08-25

**Authors:** 


### The Hospital.

### Nursing Section.

Contributions for this Section of "The Hospital" should be addressed to the Editor, "The Hospital" Nursing Section, 28 & 29 Southampton Street, Strand, London, W.C.

No. 1,040.—Vol. XL. SATURDAY, AUGUST 25, 1906.

### Notes on News from the Nursing World.

### CONDITIONS OF NURSING IN GERMANY.

To the nurses who complain of their hard lot in English hospitals or Poor-law infirmaries we commend the article which appears on another page from the pen of an English sister who has spent a great deal of time in Germany as a nurse. In one German institution, our contributor states that a nurse was made to clean windows for six months, and in another the night nurses in the male wards are not allowed to sit down all night. To add insult to injury, in the latter case all seats in the wards are removed when the night nurses appear on the scene. Worse still than either of these, the off-duty time of two hours daily is often a farce, or can only be taken at the cost of loss of reputation for earnestness. In fact, though we do not agree with all the views of our contributor, especially on the subject of friendships in hospital, she supplies ample reasons for the increasing scarcity of nurses in Germany. She also accounts for the increasing number of German nurses in England and in other countries where the rules are less stringent.

### HOSPITAL NURSES AND VIVISECTION.

Ridiculous prominence has been given in one of the daily papers to the so-called "Confessions of a Surgical Nurse," who, in the course of her "revelations," alleges that "a large number of operations take place in our hospitals which really amount to nothing less than vivisection." This statement has evidently afforded the utmost satisfaction to the anti-vivisectionists, one of whom "thankfully" cites it in support of her belief that "a vast number of unnecessary surgical or vivisectional experiments are performed on human beings." We call attention to the matter, however, only in order to protest against hospital nurses engaging in a controversy from which it is far better that they should hold entirely aloof. They can neither benefit their patients nor themselves by taking part in it.

### ESPRIT DE CORPS.

A question of great interest is raised by a correspondent who asks if nurses are loyal to each other. Having protested, not without reason, against the sweeping charges which are sometimes made against nurses as a class because of the evil-doing of a few, "I. B." goes on to answer her inquiry in the negative. We should like to have the opinion of other correspondents on this subject. There is no doubt that, as "I. B." herself suggests, a nurse is often very loyal to her training school. But loyalty to a training school is not, of course, sufficient. We do not say that nurses should under any circumstances stand by each other. There are occasions when it is absolutely necessary not to do so, but they are few and far between, and nurses should always be ready to help each other. This is the only effective means by which the spirit of *esprit de corps* can be maintained, and unless or until the maintenance of that spirit is characteristic of nurses individually, the whole body must suffer more or less. Here, however, it is character, not capacity, to which we must look for an improvement in the position.

### PROPOSED CUBICLES AT PRESTON.

The Preston Guardians last week considered a recommendation of the special committee, who advised that further accommodation should be afforded for the nursing staff in the north end of the east wing of the infirmary, and that cubicles be provided for their use. The recommendation was practically agreed to, but we hope that it is not too late to protest against the provision of cubicles. Such an enlightened Board as the Preston Guardians should hardly need to be told that nurses require a separate bedroom, and that, while it is bad enough to perpetuate cubicles, there is no defence for introducing them in the shape of fresh accommodation.

### A VISITING NURSE APPOINTED BY A TOWN COUNCIL.

At the last meeting of the Cambridge Town Council it was proposed that the Education Committee should be authorised to secure the services of a visiting nurse at a salary of £90 a year with outdoor uniform and an allowance of £5 per annum for a bicycle, the appointment not to be subject to the approval of the Local Government Board. It was stated that though the Local Government Board would not formally sanction the appointment, there was every reason to believe that they would not raise any question as to the payment of the visiting nurse. It was also stated that the chief duty of the new official would be the prevention of the spread of infection in cases of non-notifiable disease. The proposal was adopted by only a majority of one, opposition being chiefly raised on the ground that the work of a visiting nurse could be done by the two visitors selected by the Health League; and to this objection it was replied that the Health League visitors are already occupied with the prevention of infant mortality. The result of the experiment will be watched with interest; but as the Local Government Board have lately intimated to the Molesey District Council that they must persist in their refusal to approve a similar payment, we doubt whether the Cambridge Corporation will be treated differently.

### AN INTERESTING APPOINTMENT.

Many who have read the sympathetic and winning addresses of Canon Holmes to nurses will be interested to learn that Queen Alexandra has nominated him to the Mastership of St. Katharine's Hospital, Regent's Park. The choice is all the more noteworthy because it is the only piece of ecclesiastical patronage possessed by Her Majesty. Canon Holmes has relinquished the pleasant living of Sonning in order to devote his whole time to his new duties. He is a preacher of originality and eloquence, and has proved that he understands, in a remarkable degree, the peculiar difficulties and temptations to which nurses are liable.

### THE NIGHTINGALE FUND.

The report of the Nightingale Fund for 1905, which has just been issued, shows that the number of probationers admitted up to December 25, 1905, was 84; that 39 were discharged as unsuitable or left from other causes; and that 45 completed their probationary year's training and were taken on the staff as extra nurses. There remain in the Home 51, the same number as on December 25, 1905. As usual, valuable first appointments were obtained during the year by nurses who had completed their training, and also by nurses who were trained at St. Marylebone Infirmary in association with the Nightingale school. The financial statement shows a credit balance of £482 18s. 10d., the expenses including £88 as a contribution to the expenses of the training school at St. Marylebone Infirmary, and £56 gratuities to certified nurses in the same institution.

### BRAVE BELFAST NURSES.

A fire which broke out at the Throne Hospital, Belfast, on Saturday afternoon, might have had most disastrous effects if the nursing staff had not manifested great courage and presence of mind. The building is used as a convalescent home for patients at the Royal Victoria Hospital, and the room in which the fire originated contained about thirty children, many of them temporarily cripples. The city fire-brigade were promptly telephoned for, but the nurses set to work to rescue the children with such coolness and resolution that, in spite of the progress of the fire, every inmate in the hospital, adults as well as children, had been removed to a place of safety by the time the brigade arrived. Several of the children were carried through dense smoke, and sparks of fire were constantly flying about.

### THE LATE MATRON OF THE BUCHANAN HOSPITAL, ST. LEONARDS.

At a recent meeting of the Board of Management of the Buchanan Hospital, St. Leonards, a resolution was unanimously passed intimating their desire to place on record their very high appreciation of the character and work of the late matron, Miss A. C. Ransford through fourteen years of loyal service, and their deep sense of the loss which the hospital has sustained by her death. The resolution affirms that her firmness as a disciplinarian, coupled with her tender-heartedness, won the esteem and the affection of those who worked under her, and it is the opinion of the Board that to her is owing, to a large extent, the high tone which has marked the nursing staff of the hospital. Her skill and carefulness as a nurse, it is added, caused her to be loved by the patients, while the regard felt for her by the inhabitants of the town was abundantly testified by the respect and sorrow displayed by persons of all classes on the occasion of her funeral. Miss Ransford, it will be recollected, was the victim of a motor accident at the end of June.

### GALLANT RESCUE BY A SOUTHPORT NURSE.

A nurse attached to Southport Isolation Hospital, who was bathing at Douglas, Isle of Man, the other day, was instrumental in saving the life of another bather, who had got out of depth and was in difficulties. Seeing that she had been seized by cramp, Miss Wright swam out to her, held her head up, and carried her to the bathing tent, where first aid was rendered until a medical man arrived. A second nurse from the same hospital assisted Miss Wright, and the rescue was witnessed by a crowd of visitors on the promenade and beach.

### QUEEN ALEXANDRA'S IMPERIAL MILITARY NURSING SERVICE.

Sister C. Anderson has been transferred from the Military Hospital, Middelburg, Transvaal; Miss M. H. Cachemaille has been appointed as staff nurse to the Military Hospital, Portsmouth; Miss F. J. Mitchell to the Royal Victoria Hospital, Netley; and Miss J. H. Congleton to the Queen Alexandra Military Hospital, Millbank.

### THE LONDON ASSOCIATION OF NURSES, LIMITED.

Commenting on the statement that the directors of the London Association of Nurses, Limited, which has just been registered with a capital of £770, are to receive £450 per annum as remuneration, the *Financial News* says "that on the same basis the directors of a company capitalised at £1,000,000 ought to get about £580,000 a year as fees."

### NEW HOME AT WEST BROMWICH.

A Nurses' Home has just been opened in connection with West Bromwich District, Hospital which will provide accommodation for a staff of twenty-four. The cost of the building was £3,400, of which £1,168 has already been promised or paid.

### SHORT ITEMS.

Under the will of the late Mr. William Rae, L.R.C.P., the A.C.S. surgeon to the Royal Victoria Dispensary, Northampton, the Northampton Town and County Institute for Trained Nurses, benefits to the extent of £5,000, the income to be applied to the service of trained nurses in the borough. The value of garden fêtes at this season of the year in aid of nursing organisations is attested by the fact that the Wiltshire Nursing Association has benefited to the extent of about £60 by one of these functions held this month at Grittleton House.—Miss Florence Newton, formerly matron of the Wolverhampton Eye Infirmary, was married to the Rev. A. H. Lanfear at Christ Church, Wolverhampton, on August 8, on which occasion she wore the valuable diamond and pearl pendant and chain presented to her by the committee.

### The Nursing Outlook.

"From magnanimity, all fears above;
From nobler recompense, above applause,
Which owes to man's short outlook all its charm."

### A GENERAL NURSING COUNCIL.

Last week we showed how nursing had gradually developed in the last forty years; some of the effects of the substitution of better educated women for the old type of nurse; the claim of the advanced or radical party of nursing opinion to direct representation, and the practical rejection of this claim by the medical profession as represented by the resolution passed at the representative meeting of the British Medical Association held in London on July 27. In brief the evidence we adduced showed that the medical profession claim to have representation on the General Nursing Council to the extent of at least one-half of the number of members of that body. Seeing that the Select Committee, whose recommendations the British Medical Association have approved, advocates the representation of the nurse-training schools, of lay opinion, and of the nurses, it must be evident to the meanest intelligence that, if the medical profession is to have half the representation, the remaining half must be divided amongst the three other interests, in some proportion yet to be determined, and that in any circumstances the number of direct representatives of nurses on any such Council cannot exceed one-third of the whole, if they even exceed a quarter of the total members. It would thus appear that the medical profession is distinctly opposed to the views of the radical section of nursing opinion, and that it will use its influence in Parliament and elsewhere to oppose those views and to defeat them. The duties and powers of any General Nursing Council must include regulations for the issue of certificates, and the admission of trained nurses to the register, including such nurses in practice as trained nurses at the date when the act became law; regulations governing the course of training and the details and limitations of examinations for certificates; a system to control, inspect, and supervise within certain clearly defined limits the practice of registered nurses; the appointment of examiners and inspectors of nurse-training schools and examinations; the issue and cancellation of certificates; the suspension or removal from the register of the name of any nurse for breach of rules and regulations, or for conduct disgraceful in a professional sense; and the re-admission of any such nurse to such register. It further is necessary that the General Nursing Council should regulate, supervise and restrict, within due limits, the duties of nurses, and that they should regulate, and, if the promoters have their way, even control the issue of certificates and the course of training of all nurses, whilst it shall have complete control over all examinations and examiners, including the remuneration of the latter.

Any one who gives a little thought to the duties just set forth must perceive that, assuming the appointment of a General Nursing Council, it is essential that every member, whatever interest he or she may represent, shall be in a position to give many hours, and probably whole days in every week, to the adequate discharge of the duties devolving upon a member of the Council. In such circumstances how is it possible for a nurse who has to earn her own living by her profession to afford the time or to supply the means to enable her to become a working member of this body? Further, what qualifications can an ordinary nurse adduce, which shall specially fit her to regulate the course of training, the conduct of examinations, the remuneration of examiners, the inspection and control of nurse-training schools, and many other responsible and highly technical duties which will devolve upon the General Nursing Council? We make bold to say that the more nurses examine for themselves the work which any such Council will have to do in the interests of the whole of the working nurses of this country, the more they will come to the conclusion that their best interests will be served by electing representatives who possess the necessary qualifications, but who will probably be found for the most part to belong to the medical profession, to the active managers of the greater hospitals and nurse-training schools, or to have associated themselves for years with the work of other important interests which have a direct bearing upon nursing in one or more of its many and varied aspects. The point is that if Parliament were to enact to-morrow that the General Nursing Council should consist entirely of working nurses it would be practically impossible to find a sufficient number of qualified women to undertake the duties efficiently which must necessarily devolve upon any such body.

We have refrained from continually agitating the question of the registration of nurses. It is a many-sided question, surrounded by difficulties, and the time is not ripe, nor are the present circumstances of nurses such as to make it possible for Parliament to set up a system of nurse registration which shall be placed entirely in the hands of trained nurses. The London Central Hospital Council has prepared a Bill offering an alternative to registration in the shape of an official directory. The control of this directory is to be vested in the hands of the Privy Council, or the Official Registrar, or some body constituted under its authority. Seven years ago the experiment of an official directory of nurses was made at considerable cost by an independent committee of practitioners and matrons, and that publication has been continued in subsequent years, and contains the names of some 8,000 nurses. In the result it would appear that very little interest attaches to an official directory of nurses, and that there is little or no demand for such a publication.

### Abdominal Surgery.

By Harold Burrows, M.B., F.R.C.S., Assistant Surgeon to the Seamen's Hospital, Greenwich, and to the Bolingbroke Hospital, Wandsworth Common.

### Uterine Fibroids.

Fibroids or fibromyomata of the uterus are non-malignant tumours, which usually first give rise to symptoms in early adult life. The chief and earliest sign is hæmorrhage. This may be merely an excessive amount at the normal periods (menorrhagia), or it may appear in the intervals also (metrorrhagia). If allowed to run their course, fibroids may form large abdominal tumours and produce severe symptoms by the pressure they exert on neighbouring structures. Thus they may cause pain, constipation, and excessive frequency or difficulty in micturition. In long-standing cases profound anæmia may ensue upon the repeated loss of blood.

Uterine fibroids are subject to certain special complications; the most important of which are infection, sloughing, and cancerous degeneration. Sometimes, as the patient becomes older, the fibroids cease to grow, and this arrest is the more probable when the patient has passed the climacteric.

The treatment of fibroids may be palliative or operative. Palliative treatment includes rest in bed at the menstrual periods, or whenever the hæmorrhage is severe, with the administration of ergot and iron to counteract the bleeding. At the same time a careful watch is kept for the appearance of marked anæmia, pressure symptoms, and other complications, and the size of the tumour is noted from time to time. If the hæmorrhage or pressure effects are so severe as to render the patient miserable or to endanger her life, or if the tumour is growing rapidly, the condition ought to be treated by operation. And, as in most surgical complaints, the sooner the operation is performed the better is the prospect of a good result.

The operations that are undertaken for fibroids are (1) removal of the tumour alone (myomectomy); (2) removal of the uterus (hysterectomy); (3) removal of the ovaries and tubes. The last named is not often performed; its object is to produce an artificial menopause, and so to arrest the hæmorrhage and check the growth of the tumour.

### Cancer of Uterus.

Cancer of the uterus is most common in women who have passed middle age. In the early stages the leading, and perhaps the only, symptom is hæmorrhage. It should be a rule always to regard irregular uterine hæmorrhages with the gravest suspicion. The bleeding, however slight it is, always indicates the presence of mischief, which it may be fatal to neglect. In patients past the menopause there is an unpleasantly strong suggestion of cancer in all such cases.

If the hæmorrhage is ignored and the case allowed to go untreated, other symptoms will soon arise if cancer is present. These are pain, foul vaginal discharge, loss of appetite, wasting, anæmia, weakness, and general ill-health. Still later, fistulæ may open into the bladder and rectum, and the patient's condition may soon become deplorable.

The only treatment which has any chance of curing the patient is to remove the uterus (hysterectomy) in the earliest stage of the illness. By the time that advanced symptoms, such as pain, foul discharge, and constitutional disturbance, have appeared, the favourable opportunity has been lost. Hence it is silly in the extreme to ignore irregular uterine hæmorrhage, and to defer seeking advice and treatment until compelled to do so by the appearance of some disastrous complication. In the late and inoperable stages of uterine cancer the treatment consists chiefly of frequent douching to clear away septic discharges and to diminish hæmorrhage, and of efforts to relieve pain.

Unfortunately there is no satisfactory method of relieving pain in a case which is likely to continue over a long period of time. Morphia, which is such a divine remedy in acute cases, must be administered with great caution to the patient who has cancer, unless she is near the end of her illness. For it is apt after a while to take away the capacity to bear pain; so that the patient no longer has the courage to brave out her affliction, and her misery becomes extreme.

### Affections of the Fallopian Tubes.

Each of the two fallopian tubes opens at one end into the uterus, and at the other end into the peritoneal cavity, close to the ovary.

Microbic infection sometimes spreads to the tubes from the uterus, the latter having been infected from the vagina.

When this has taken place the tube becomes swollen, inflamed, and tender (salpingitis), and in the course of time the infective process is apt to spread to the peritoneum. Having got so far, the

The Area with Cross-hatching shows the Situation of Pain and Tenderness in Pelvic Peritonitis.

micro-organisms may set up general peritonitis, but more commonly the peritonitis remains localised to the pelvis.

Pelvic peritonitis originating in this way is a fairly common ailment among young women. Its course is variable. The inflammation may subside, leaving no clear trace behind. In other cases a pelvic abscess forms. In most cases there remain adhesions which interfere with the functions of the pelvic organs, causing chronic pain, dysmenorrhœa, constipation, and other troubles, sometimes so severe as to lead to chronic invalidism.

The symptoms in the acute stage are those of a local peritonitis. There is severe pain in the lower part of the abdomen, the pulse-rate is quickened, the temperature raised, and the patient may vomit. There may be increased frequency of micturition, and pain during the act, and defæcation, or the administration of an enema may cause much distress. Sometimes the patient complains at first of general abdominal pain, and in such a case, unless the history suggests a pelvic origin for the complaint, it may be very difficult for the surgeon to decide the right treatment. The presence of a vaginal discharge is an important clue in such a case, and if the nurse discovers such a discharge or is aware that there has been one, she should not fail to report the fact to the surgeon.

The treatment of pelvic peritonitis in the acute stage usually consists of rest in bed, light diet, vaginal douches, fomentations to the abdomen, aperients, and sometimes opium to relieve the pain. If an abscess forms, it will need to be opened, and if one or both the fallopian tubes remain swollen and tender (pyosalpinx, hydrosalpinx), and pain and other symptoms continue, it may be necessary to perform a laparotomy and remove the affected tubes.

### The Nurses' Clinic.

### CONSTIPATION AND ITS CURE.

There can be but few nurses who have not discovered during their professional life how widespread is the evil of constipation among women. We hear of it so constantly that it seems as if 50 per cent. of the women we come in contact with are suffering more or less severely from this ailment. Various forms of medicine are in daily use by a certain class of patients, perhaps patent pills are the favourite remedy, and these are generally taken without medical advice; but, as we know, strong physic only serves to increase the evil, and the unfortunate patient goes on increasing the dose with disastrous results to the delicate lining of the internal organs affected and with absolute failure in the matter of curative effect. Nurses, who know that long continued constipation brings other evils in its train, may do much to counteract the habit of constant physicking as a cure for constipation. They can gently point out the dangers of this habit and indicate a better and less injurious way of getting relief from this all too common ailment. Various reasons may be given to account for such a widespread evil. Many women are ignorant of the value of simpler treatment or they dislike the trouble of persevering in a course of special diet, or they are unable to take sufficient exercise, or possibly they have never established regular habits in this direction. Whatever be the cause, however, the fact remains that women suffer greatly both from the evil itself and from the means they use for its cure. So great is the injury done to other internal organs by the pressure of over-loaded bowels, that it is worth while to consider what means may be adopted to avoid such injury in a natural and harmless way.

Much may be done by starting a simple course of diet, which must be persevered in for some months at least. The writer has found much benefit from the use of Bermaline or malt bread instead of the white loaf, from which is eliminated a laxative element of great value to the human body. Another item of importance is the consumption of more fruit in the daily diet, especially at breakfast time. In obstinate cases fruit eaten immediately upon waking is very beneficial, such as a baked apple, a stewed pear, an orange, or stewed prunes or figs. The latter have a specific laxative effect, and are, moreover, very agreeable to the taste. Marmalade should always find a place upon the breakfast table, likewise oatmeal porridge, to be eaten preferably with golden syrup. Clotted cream and gingerbread are also good laxatives. Many people find a glass of cold water taken in the early morning very effectual. Those who have the care of children and young folks will find the above suggestions particularly helpful if regularly carried out.

For adults, treatment by diet may be supplemented by simple physical drill which should ensure the exercise of those muscles not brought into use by the ordinary movements of the body. This form of treatment is especially called for in the case of persons who lead a sedentary life or who from various reasons do not take sufficient exercise in the open air. The old-fashioned dumb-bells might be revived with great benefit to lethargic, phlegmatic temperaments. The liver becomes sluggish for want of vigorous movement on our part, and a sluggish liver means a good deal of general bodily disorder. There is a great deal of truth in the new Gospel of Nature-Cures that we hear and read so much about in these days. With a little help from ourselves, Nature will work wonders; and to a very great extent we may work out our own salvation as far as health is concerned.

As regards nursing treatment, gentle massage of the bowels is useful, also a cold-water compress placed upon the abdomen at night and secured in place by a firm binder. A vigorous sponging of the abdomen with cold water in the morning, to be followed with an equally vigorous rubbing with a rough towel, is often very efficacious; but in following this treatment care should be taken to let the movements follow the course or direction in which the food travels through the intestines in its onward path towards the rectum.

Sometimes the patient's medical adviser will order an enema to be administered. This will generally be a soap and water enema, or an enema of warm olive oil, or a glycerine enema. The latter usually acts with great promptness, and the patient can learn to use it for herself. In every case the nozzle of the syringe must be well oiled so as not to irritate the delicate passage into which it enters, and care must be taken that no air enters at the same time as the fluid which is being injected.

Sometimes glycerine and castor oil in equal parts are very useful. Whatever be ordered, the fluid should be warmed to the temperature of the body, and violence must never be used in introducing a nozzle. No patient should be left long alone after the administration of an enema, as sometimes faintness comes on, and as soon as possible a warm comforting drink should be given. For persons who suffer from constipation coffee is preferable as a drink to tea, the latter having strong astringent properties. A sparing use of meat is also advisable, but a liberal use of fat of all kinds may be strongly recommended. In the case of

**THE NURSES' CLINIC—*Continued.***

young children and infants, the finger of the nurse, well oiled or smeared with vaseline or common yellow soap and passed into the rectum will be all that is necessary to effect an evacuation. Children should always be encouraged to form regular habits of body so that trouble may be saved in after years. It is perfectly amazing to see the array of medicine bottles in the bedrooms of many girls and young women. They are shy of seeking medical advice on such a subject and start doctoring themselves with astonishing confidence, and very often the mischief is thoroughly deep-rooted before any advice is sought, and then, of course, it is more difficult to cure the ailment. A kindly nurse can often win a patient's confidence, and by the exercise of common sense and tactfulness be of the greatest help and assistance to suffering womankind—and who could desire a more useful rôle in life?

### Incidents in a Nurse's Life.

### GETTING RID OF THE PATIENT.

I was quite a new probationer, and had been much interested in a case of attempted suicide, which the patient had endeavoured to effect by taking turpentine; she was a most obstinate girl, and sometimes refused food, and often gave us a good deal of trouble. One day Clara was told she could get up, and that same evening Sister said to me, "Nurse Mary, I want you to take Clara home, be ready to start at seven o'clock." I felt most important, and put on my bonnet and cloak at the appointed hour. Just as we were starting, the house surgeon called me. "Whatever you do, nurse, you are not to bring her back," he said. I began to see trouble ahead; however, we drove off in pouring rain, and after about half-an-hour pulled up at a villa with one very dim light in a lower window. I helped Clara out, and, leaning on my arm, she walked up a path with many shrubs on one side. I pulled the bell, and soon we heard little pattering feet and a small voice said: "I can't let you in." I explained that I had brought the girl back. "Mother said I was to open the door to no one," was the reply. Then Clara spoke, "It is Clara come back, let me in, Master Harold," but there was no response, and shortly we heard the little boy saying as he ran along a wall near by, like a cat, "I am going to fetch mother, she is up at grandma's." "Where is that?" I asked, and, to my dismay, Clara answered, "A long way off." I did not know what to do; I had been told not to bring the girl back—and I was terribly afraid of the house surgeon—and here was my patient shivering in the rain and not long out of bed. "You leave me, nurse," she said more than once, which, of course, was impossible. A few minutes later we heard a rustle in the darkness and a servant rushed hastily by us. "Come, Clara," I said, "we will follow her." We passed through a door into another garden, and stumblingly tried to pursue the shadowy form in the darkness, but we heard a door banged and a key turned in a lock, and knew that we were left outside.

Again I rang the bell, and had the same answer from the servant, "I am not to open the door to anyone." Then we went to the cabman; he thought we had better sit in the fly, and he would watch the gate and tell me directly anyone entered it. Before long he came and, in quite an excited manner, reported that a man had gone through the gate. Out we got again, and once more I dragged poor Clara up to the front door. I rang the bell and waited; I could hear a man's steps pacing up and down the hall, but no one came to the door. I felt utterly hopeless, and began to imagine all sorts of dreadful things. For one thing, I was haunted with the dread that my patient would probably die of pneumonia from exposure; she was but thinly clad, and the wind and rain were fighting against her.

Then I heard footsteps, and a young man appeared close to us, looking very confused and pulling his moustache. "She cannot be taken in, nurse," he began, when I eagerly interrupted. "But I have been told distinctly to leave her here." "I have nothing to do with the girl," he said, "but my friends wish me to say that on no account can she be received into the house again." "And I cannot take her back, sir, for I was particularly told not to do so." "You can take her to the workhouse, then." "I was told to leave her here; I am only a new probationer, and I cannot tell you how much I shall get scolded if I take her back to the hospital, and certainly I cannot take her elsewhere, without the house surgeon's permission. "I am very sorry for you, nurse," the young man said, "Dr. —— ought not to have sent the girl at all; he knew we could not have her back again." For about ten minutes we argued, for I was determined not to give in, but ultimately I had to do so, and feeling most uncomfortable I placed Clara in the cab again, and all the way back I wondered if she would strangle me or try to jump out of the window. However, she behaved very well, and as soon as I had taken her to the ward again, I went in search of the house surgeon. I told my story, and he listened very attentively, merely saying as I finished: "Well, nurse, you have done the best you could, get the girl to bed as soon as you can."

The next day Clara was sent to the Workhouse Infirmary.

### The Red Cross Societies' Exhibits at Milan.

BY OUR OWN CORRESPONDENT.

One of the most attractive sections in the great Exhibition now open in Milan is that devoted to ambulance and hospital work, as carried out under the jurisdiction of the various national Red Cross Societies. The British Red Cross Society takes no part in this, because the Red Cross Society in this country occupies a quite secondary and supplementary position, the Army Medical Corps and the Admiralty having their own full equipment for war or peace. Nor does the British Red Cross Society assume any responsible part in civil work, which is amply covered by the Board of Health departments, the St. John Ambulance Association, and local bodies. On the Continent all these varied duties, both military and civil, are brought within the Red Cross sphere, if not actually undertaken by the Society. It is impossible and useless, therefore, to make comparison between the work of these societies and our own. It may, however, be both possible and profitable to study the work that is done by the foreign organisations.

The Italian Red Cross Society (*Croce Rossa Italiana*), in a popular memorandum which it circulates, deplores its lack of support, comparing its position with the more favoured treatment accorded by other nations to their own similar societies. There is undoubtedly some truth in this, yet the Italian Red Cross work is excellently well done, despite lack of funds. Since it first began operating, immediately after the rules of the Geneva Convention had been drawn up, its

### THE RED CROSS SOCIETIES' EXHIBITS AT MILAN—*continued.*

work has extended rapidly, and although care for the sick and wounded in time of war, and the maintenance of military hospitals in time of peace were its first development, it now informs its supporters that its activities extend over a still wider area in civil life. Its task is one that is continually growing heavier. It has to maintain stations for first aid as well as an ambulance service; it has depots established close to the pits where mining is proceeding, especially in Sicily, where the sulphur mines are dangerous, and also it has undertaken an energetic campaign against the *Agro Romana*—the malarial scourge. All this means, of course, a vast organisation spread like a network over the country. It is of special interest, therefore, to note some of the methods and some of the tools employed by the Croce Rossa in its endeavour to meet its responsibilities.

What impressed me personally, in looking at the various exhibits made by the Italian Red Cross Society, was its excellently equipped service of barge boats, or *Ambulance Fluviale.* At least three of these are shown at Milan. Outwardly the barges look very ordinary, but their interiors have been transformed into one or two (in one case into three) "saloons," fitted with berths arranged in tiers, with cabin for the medical officer, bed for the attendant, large kitchen fully fitted, and a *farmaccia* or laboratory. The

A Room in the Italian Red Cross Hospital Train.

berths are generally canvas stretchers on stout poles, and so fixed that they can be lifted down by bearers and carried in or out of the cabin with the patient lying down. They are furnished with thin mattress, pillow, sheets and blanket, and scarlet rug. The white and red combine with the green of the canvas awning to make the Italian national colours. The ventilation is good, the absolute simplicity of the fitments and their character makes cleanliness assured, the water supply is obtained through Berkefeld filters, and heating in cold weather is obtained by the use of porcelain stoves. Altogether these floating infirmaries are pleasant places.

The Red Cross Hospital Train is well arranged and apparently should answer every need, although the Italian type does not supply such elaborations as the German Red Cross Society displays. The kitchen is placed in the centre of the train, and has a double range with boiling apparatus and steamer-pans. The ware is either block tin or enamel; there are cabinets fitted into corners and folding tables, lockers, and refrigerators. The larder and farmaccia adjoin the kitchen at its respective ends, then the next wagons provide cars for the sick, with a special small one for the surgeon who travels with the train. Transport surgical tents, with operating-tables, chairs and all apparatus, are made portable in sections. A speciality is also made by the Italian Society of wicker and rope cradles for conveying the wounded up from pits or from wrecks, and one noticed as well the straw pads for temporary service as splints, and light iron frames to support broken limbs.

Italian ambulance wagons for service in towns are largely provided with electric traction, electricity being much more commonly employed in Italy for traction purposes than it is with us. In Naples there are electric hospital trams, for conveying the sick through the city, from one hospital to another, and possibly for use as a means of giving convalescents an airing, as they are made with curtained sides for summer service.

The German Hospital Train, as already indicated, is almost luxurious in its fittings; there are camp chairs by the side of each berth, the latter are also of a superior type and have a bed-table attached, and a rack above to hold extra wraps or papers, also shades to the windows and lamps. The ventilation is provided by gratings along the central corridor, and the floor is covered with cork carpeting. There is an operating-room, as well as the surgery, added to each train, and a very fully equipped kitchen and larder.

The Swiss Red Cross Society makes a larger display than any other nationality, but chiefly in models of sanatoria and of hospitals in its different cantons. Considering how many of these buildings are situated high up among the mountains, it is interesting to notice the carrying provision for the sick. Some appear to have a cradle of wicker, or a light wooden frame; others are in the form of a sledge or a barrow with long handles; most of them are padded or have mattresses and canvas coverings. There are Samaritan stations in different places, with hospitals for spinal diseases, throat and nervous disorders, as well as for the treatment of tuberculosis patients. A large exhibit is made of hospital fitments by the well-known firm of Hausmann, of St. Gall, Geneva, Zurich and Bale. The testing apparatus, syringes, and chairs adapted for the examination of spinal curvature, and for trying the strength and weight of a patient, seemed especially good. The study of sanitation as applied to towns, and the inspection of food and its preparation is a section which is in Switzerland given up to the White Cross Society, and a particularly strong branch of this society has its headquarters at Berne. These societies have sent up volumes of reports dealing with the sanitary inspection of public buildings, hospitals, and sanatoria in every district, and a look into some of them would be very helpful to a student preparing for work as sanitary inspector. There are likewise charts detailing the number and kind of accidents in factories and industrial centres, according to character of the trades concerned. Metal-workers and machinery makers furnish the record number, but the proportion of accidents occurring in the pottery trade is also high. Switzerland seems, indeed, to carry the study of hygiene much farther than is the rule in other countries, but this is natural considering that she is the receiver of the sick of all nations, who fill her hospitable and well-kept cure-houses.

Although I made no notes of anything of special import in the small exhibit of the Japanese Red Cross Society, it would only be fitting to quote here the judgment which Sir Frederick Treves has passed upon the work done by this association, after he had personally examined it at first hand. He says:

"This business-like organisation is the most remarkable and efficient of its kind in the world. It not only undertakes to look after its sick and wounded, and so relieve the War Office at a critical moment of responsibility, but it concerns itself with the soldier's comfort from the beginning of the campaign to the end. It "mothers" him in a sensible manner, without either extravagance or hysteria, and makes him feel that all that is done is merely the endeavour of the country to show its appreciation of his services and its sympathy with his hardships. The society continues its work when the war is over, and does not depend for its maintenance upon a fitful and ecstatic outburst of sentiment, which barely survives the crisis which evoked it. This is the noble feature of the Red Cross Society of Japan, that after the glamour has faded the soldier is not forgotten."

### Nursing on the Borderland of Afghanistan.

In describing my work in a hospital in Beluchistan I will not speak much of the hospital work, for in many ways it is much the same as that so ably described in a recent issue of the *Hospital* "Mission Nursing in India." Suffice it to say that here, on the borderland of Afghanistan, we suffer a good deal from the extremes of cold and heat. In the winter it is very cold, my room rarely getting above 30° F. when the sun is down; whilst in June it is often 90° F. with every window and door open. We have, however, the great comfort of cool evenings and invariably get a breeze of some kind. Quetta is surrounded by huge mountains, and when the thunder rolls around it is truly awe-inspiring. Last night we had a "wind storm," and as we could not keep a lamp alight while I went my round with the night nurse, we waited for the lightning flashes and by this means saw each patient clearly. Here we have a mixture of three languages to face, and also a curious medley of tribes, Brahmi, Pathan, Hazari, Hindu, all coming daily to our hospital for treatment. On most days the Gospel message is given in the three languages. But my work lies a good deal amongst the patients in their own homes and the curious methods they pursue for the alleviation of pain are most interesting.

### The Men who Came to Mourn.

One very cold wet night in early January, a man came about 10 p.m. He was in great distress about his wife. She had been ill for days, and he was sure if the "Mem Sahib" would but look at her she would get well; I got my bag and entered the "Tam Tam" (native carriage) with my interpreter, and also, I admit, very many misgivings as to the effect "a look" from me would produce. After a lot of plunging through streams, etc., we reached the house, or rather room, for the other room was occupied by cattle. There were two beds in the room, the length of which was about 16 ft. by 5 ft. On one bed squatted about were eleven men, on the other all I could at first discern were two thick-padded native quilts. After turning the men out I found my patient nearly smothered between the quilts, a brief examination revealed a rather severe case of hæmorrhage, and on giving the usual treatment we returned home, leaving strict orders that only the husband was to remain with the patient. The poor men looked rather troubled, for they had "come to mourn awhile," which the English "Mem Sahib" did not approve of, for the atmosphere was almost like that of a "London fog."

### A Midwifery Patient.

My next interesting case was a midwifery one. My native dai (midwife) called me to a case one afternoon, saying "there are some untrained dais in the house, and if you don't come they will give her fever." Off I went, stumbling over one woman on entering the wee room and nearly falling into a small "augithi" (charcoal fire). There was no window of any description in the room, only one brick had been left out high up on one side of the wall; this and the door were the only methods of ventilation! The women fled on my approach, muttering loudly. The poor patient was screaming and evidently in great terror as to what I might do. First I procured a lamp. This was only a native lamp, the small clay saucer affair one sees at home in exhibitions, with a little rolled cotton floating in the oil and allowed to hang over one side, the hanging portion giving forth light, or at least supposed to do so. I made inquiries for hot water and some cloth to wrap the prospective baby in; on examination I found there was no time to lose, and almost before we were aware he had arrived, and was apparently stillborn.

### The Baby.

The trained midwife attended to the mother while I quickly proceeded with artificial respiration, calling for hot and cold water; the former was brought in in a kerosene oil tin, the latter in one of the usual flat earthenware dishes, very like the old-fashioned butter-dishes used in some of our farmhouses in England. I had to double baby up to dip him in the hot water and unroll him for the effect of cold, but, wonderful to relate, he at last cried. Then I proceeded to dress him: not a rag of any kind could be obtained, so in desperation I made the husband remove his turban. As it was a male child I could dare to ask for anything. Fortunately, too, the turban was made of woollen material, and I made it act as binder and bara and dress. There was plenty of it as these turbans are three or four yards in length. I now took the baby to the mother, for, of course, I was anxious that he should be kept warm; but imagine my disgust when I learnt that "she could not have him near her until the star came out in the east." This is a custom which no amount of persuasion will overrule; so I gave the wee man a warm corner near the fire and left.

### Native Treatment.

Another amusing case came the same week, and the methods used here to produce quick delivery might be useful to my sisters at home! This was a prima-para and a Hazari woman. I was called out about 7.45 a.m. Upon examination I determined that it would not be advisable to leave the case, as I had some idea of what might occur in my absence. Therefore, as my language is limited—I only came out in November—I contented myself with sitting down, keeping my eyes open lest they should put my patient in danger, but at the same time not interfering because I was earnest in my desire to see their methods. First, a large handful of flour was placed on a plate and waved over her head three times, then some live charcoal in a small augithi likewise passed over. This was taken away and small flat cakes made, they were then brought in and broken over her head. Still the pain continued slow, so six or seven rupees (native coins about the size of a florin) were wrapped in rag and tied around the right arm of the patient, also the hem was torn off a little girl's gown and tied around the waist; strange to say this, too, proved of no avail!

### Killing a Fowl.

Then I heard them say "bring a fowl," and I moved to a discreet distance. The fowl was brought by the husband, who

NURSING ON THE BORDERLAND OF AFGHANISTAN—*continued.*

immediately made an incision in the neck, and while the warm blood flowed it was placed in the form of a circle on the woman's forehead, then sprinkled all around her on the ground. The native women, I ought to explain, always sit on the floor of their houses until baby arrives. But this fowl remedy also proved futile. Everything, however, in my judgment was going on all well, and as the room was rather odoriferous and the temperature about 200° F., I walked to the door for a moment; hearing a noise I looked behind to find to my horror the friends had lifted the woman up in her blanket and were tossing her to and fro like a ball. This I immediately stopped and sent out all but one woman. Then in about half an hour the baby arrived. In this instance I had procured a clean, really clean, pair of the father's pyjamas. They are made of white calico, very primitive in shape, being two huge flour bag-like legs, drawn into tight bands at the ankles. Having torn these up in readiness I was able to dress baby very comfortably and return to hospital. The natives consider it very wrong to prepare clothing for either the baby or the mother beforehand, and this, to me, is naturally a constant source of distress.

### Why Nurses are Scarce in Germany.

BY AN ENGLISH SISTER.

I have lived in Germany now for some years, and the increasing scarcity of nurses is daily becoming more apparent. Anyone carefully perusing the advertisement-sheets cannot but be struck by the number of committees seeking nurses both for hospitals and for private work.

Not only are the medium-sized hospitals in great want of more nurses, but large institutions are no better off, though they do not openly advertise—and the reasons are not far to seek. In one famous hospital a girl of good birth and education—in fact, she was a member of the German nobility—was made to clean windows for six months. The reason given for this treatment was that it would break her pride, the need of so doing being considered obvious because after three months of perpetual window-cleaning she had dared to ask if her duties were always to consist of this particular duty. At another hospital in a well-known city, the night nurses in the male wards are not allowed to sit down all night; and so, that there shall be no temptation to do so, all seats are removed when the night nurses appear on the scene.

English nurses generally have special hours on and off duty. Theoretically, it is so in Germany, but very seldom is this rule practically carried out. Nurses are supposed to have two hours off duty daily in most German hospitals, and to go out for one of these hours, but I know several different hospitals, strictly speaking "Mutterhäuser," where a nurse is, if not actually forbidden to go out for her hour, at least looked upon, if she does so, as a very frivolous worldly-minded being. The consequence is that she generally very soon gives up going out regularly at all.

Then, again, I have very often personally observed, as well as heard of, the unkind custom of constantly changing nurses. If the "Mutterhaus" sees that they are popular at the hospitals or in the parish where they work, the nurse suddenly receives a blue letter and finds that she must go on to another hospital or another parish at a couple of days' notice. I suppose the rule has its good points or it would not be so universal, but whatever its advantages may be decidedly its disadvantages are still more obvious.

It is easy to imagine how exceedingly annoying it is for the medical men who have just got their surgical nurse into their own special ways—and most doctors have some particular ideas with regard to nursing—to find that they must begin again with a new nurse, knowing also that in two years, or three at the latest, she also will be removed. Then, too, it is equally trying for the old parish patients, who are often suspicious and faddy, to find that the "Schwester," whom they often grumble at themselves, but whom they never allow anyone else to breathe even a word against, is going to leave them almost without warning. The learning up of the ways of some of the people is no sinecure, for anyone coming to Germany for the first time and visiting parish people would be astonished at the great number of pillows the poor like at night and the endless number of petticoats and wraps by day. Seventeen pillows at night is quite an average number to sleep with!

Then, in numbers of hospital one finds that almost the greatest crime a nurse can commit is to make a real friendship with another nurse—or, in fact, with anyone. As soon as it is noticed that the nurse has a friend, be she nurse or outsider, she will discover that she is treated as a very black sheep. Her letters will be outwardly if not inwardly examined, remarks will be passed, such as "What! another letter with the L. post-mark?" etc., and in every way she will feel that she is in disgrace until she either gives up all friendly intercourse, leaves the institute, or, what is more likely, becomes sly and deceitful and keeps her friend by hiding her friendship. Then, again, most hospitals have a rule that the nurse must always (without exception) wear uniform; not even during holidays may she wear ordinary dress nor may she go to the theatre or concerts, except where religious music is given, nor even to a large private party. I do know a few (a very few) large hospitals, where the treatment is more humane, and the nurses may be without uniform when on leave, and also visit a theatre, but these hospitals are still in a great minority.

As to a "Schwester" (sister) becoming engaged—and in spite of rules and hospital laws, that does happen sometimes even in Germany—it will not bear thinking of. If, however, such a very "unschwesterliche" (unsisterly) event should occur, the committee must be told at once and she must leave immediately. As German nurses are not nuns they can quit after sufficient notice—according to the different rules of different hospitals—but I do not know of any hospital where they will allow a nurse who has become engaged to go on nursing until it suits her to marry. All things considered, I do not think I am wrong in saying that, until German hospital authorities alter their very strict rules and begin to treat nurses as grown women with common sense and individuality, instead of tyrannising over them as if they were school children likely to get into mischief, the scarcity of nurses will continue.

I was brought in contact with a clergyman who was the life and soul of a large deaconess' home, who was on several of the hospital committees, and received a badge from the Emperor and from a Grand Duke for his services in aid of hospitals. He had eleven daughters, but though he was always endeavouring to persuade all the girls with whom he came in contact of the desirability of their becoming nurses, he would not allow a single one of his own daughters to embrace the nursing profession—a fact which speaks for itself!

### Everybody's Opinion.

### AMERICA AND ENGLAND.

"An American Nurse" writes: Being disgusted with nursing in England, I have come back to the States. Decidedly just now nursing in England is as bad as it possibly can get, and is sadly in need of repair. My experience is that "specialists" dress their housemaids in nurses' uniforms, and charge three guineas a week for their services, or send them as special nurses for special operations with special fees. Nursing homes, too, palm housemaids and lady-helps on to the public, who, by their vulgarity and want of training, bring degradation on the nursing profession; and the strange thing is that the trained nurses do not seem to care. Now in America a nurse strives ever onward and upward; the masses of nurses act in unison. Hence the medical and nursing profession are here practically on equal footing, while in England the doctor is Lord Pills and the nurse often treated as a servant. True, it never happened in my case, as I would never nurse for a vulgar doctor nor in the house of ill-bred people.

### ARE NURSES LOYAL?

"I. B." writes:—I was truly amazed a short while back at being asked, in all good faith and seriousness, by a lady who moves in good society if it was really true that private nurses were as a class such bad and immoral women? I answered, what I believe to be the truth, to the effect that though no doubt there were women who used their profession as a cloak for immorality, I thought they were few and far between, and that private nurses were as a rule a most persevering and hardworking class, the majority of them leading a monotonous and, to a great degree nowadays, thankless existence. To this she replied that having had an operation performed some time before, she had asked her nurse the same question, and was informed, with apparently sincere regret, that she was sorry to have to say it, but as a class she feared that what was said was only too true! I have been a member of the nursing profession for fifteen years, and a private nurse for seven; I have worked in the provinces, in London and suburbs, and abroad, and I have lived and worked among hundreds, if not even thousands, of nurses from all corners of the British Isles; yet, though many be their faults—being but human—still it is impossible to think that the one laid at their door in this instance is true, notwithstanding that many people, and alas! evidently some nurses too, would have us believe it so. Perhaps one can hardly be surprised at the judgment of the public when we remember how hard nurses are on each other. The nurse who is ill is frequently, if not generally, credited with hysteria until, perhaps, total collapse tells its own tale. Two terrible cases of this kind have occurred within my own experience, one of which terminated fatally and the other in months of illness, both nurses developing enteric and both in different hospitals. We constantly see and hear of the hospital-trained woman sneering at one with only infirmary training, and both at the nurse who has only mental or fever training, and yet the merit of the former may often rest solely on the length of her certificate. One even reads articles in which a nurse—after acknowledging that she has recovered from four serious illnesses, presumably with the aid of the nurses in question—rails at them most bitterly for spilling candle-grease and spoiling towels—according to the servants' account given, as usual, after the nurse's departure. If one goes into any other than a nurses' institution it is usual to find someone who will come forward, probably at the request of the principal, and chat and initiate the newcomer into the ways of the household, trying to make her feel at home. This is a most noticeable feature of Girls' Friendly Society and Young Women's Christian Association homes, why not so with nurses? More often than not it is quite an ordeal to have to face a roomful of nurses and not see a single friendly face, to have your remarks received in stony silence for the most part, until someone, more bold than the others, has put the question as to where you were trained. If you do not feel inclined to satisfy such impertinent curiosity, you will be dubbed untrained without a doubt and treated accordingly, until by force of character or otherwise you may win a place in their esteem. No doubt this habit of suspiciousness, or whatever it is, is acquired in probationer days, when the last new arrival must be taught her place and be made to understand that she may watch and listen but not speak until she is spoken to by her superiors in training, or she will be apt to be sat upon very promptly. After three or four years of this kind of thing it is natural that she should mete out the same treatment to others in their turn, and for the rest of her nursing career believe it to be the correct attitude towards all newcomers. A nurse may be, and often is, very loyal to her training school; why should it be such an impossibility to extend this loyalty to her fellow-nurses from all institutions? If she would once learn to back them up, all and sundry, she would soon be able to move mountains, and to solve more difficulties in a week than State Rgistration will in years.

### Appointments.

Crosland Moor Union Hospital.—Miss Mary Alice Brierley has been appointed charge nurse. She was trained at the Union Hospital, Ashton-under-Lyne.

East Riding County Asylum, Beverley.—Miss Caroline Fielding has been appointed matron. She was trained at the Infirmary, Horton Lane, Bradford, and was afterwards theatre nurse and ward sister at the same institution. She was nurse at Daneswood Sanatorium, sister at Stobhill Hospital, Glasgow, and assistant matron at Stirling District Asylum, Larbert. She holds the C.M.B. certificate.

Hospital for Epilepsy, Maida Vale, London.—Miss Mary L. Pollett has been appointed matron. She was trained at the London Hospital, and has since been sister at Radcliffe Infirmary, Oxford; assistant matron at the General Hospital, Nottingham; and matron of the Cottage Hospital, Abingdon.

Isolation Hospital, Abingdon.—Miss Frances Despard has been appointed sister at this institution. She was trained at the Blackburn Infirmary, Lancashire.

Park Hospital, Hither Green, S.E.—Miss E. L. Price has been appointed charge nurse. She was trained at the Holborn Infirmary, Highgate.

The Lady Dufferin Hospital, Karachi, India.—Miss Sara T. Sutcliffe has been appointed matron. She was trained at the Clayton Hospital, Wakefield, at the Manchester Southern Hospital for Women, and at the Manchester Maternity Hospital. She has since been ward sister and matron's assistant at the Manchester Maternity Hospital, and matron of the Birkenhead Maternity Hospital.

### Presentations.

Warrington District Nursing Association.—Miss Whitfield, superintendent, and Nurses Jacocks and Leshaw have resigned their appointments on the Warrington District Nursing Association, after about eight or nine years' service. To show how they have been appreciated the patients and friends have presented Miss Whitfield with a silver tea-set and oak mounted tray, Miss Jacocks with silver candlesticks and ink-stand, and Miss Leshaw with a case of brushes with silver backs.

Isolation Hospital, Bow Arrow Lane, Dartford.—Miss A. L. Mitchell, charge nurse at the above hospital for the past seven years, on the occasion of her marriage, has been presented with a silver tea-service by the matron and staff, with a gold brooch by Mrs. O'Neill Roe, of Iquique, the late matron, and with a case of silver fish knives and forks by the medical superintendent.

### Practical Hints.

We welcome notes on practical points from nurses.

### THE CARE OF THE FEET.

Knowing from experience how much nurses suffer from their feet, especially during the first few months of their hospital training, it has struck me that a few words on the subject may be of help. I cannot impress too strongly on nurses the fact that they must not begin by wearing new shoes. These at all times are hard and unyielding, how much more so when the unfortunate wearer has no opportunity for many hours of going to her room to change them. A would-be probationer will find it a help to rub her feet every night before going to hospital with methylated spirit or to soak them in cold salt water to harden them. A great mistake made by nine out of every ten girls in the changing the style of her footwear—*i.e.* from wearing ordinary indoor slippers with moderate heels she thinks it necessary to buy a new pair of ward shoes with little or no heel. This will alter the strain on the ankle and calf and she will suffer greatly in consequence. If she has been in the habit of wearing these shoes by all means let her continue to do so, but if not she cannot do better than wear an old pair of her ordinary shoes with rubber heels, and, if very thin, the addition of a cork sole. Very thin soles and high heels are a "weariness to the flesh"! For the first few weeks possibly the new probationer may suffer very much from hot and swollen feet. She will find it a great relief to raise the end of her bed on bricks and loosen the bedclothes. If bricks or blocks cannot be found a cardboard dress box under the mattress answers the purpose just as well.

On night duty she will be required to wear soft felt slippers and may find the same strain on her muscles—a heel pad inside the shoe will help her. I remember during my training being told by a very wise Matron always to sit down when possible, and I have made it a point when training my nurses to see that they do so when doing such work as rolling bandages, cutting dressings, padding splints, etc. I have heard nurses say they were not allowed by their Sister to sit down, but after many years' experience I have never found one who considered it a want of etiquette, and a nurse can always stand up if the Sister comes up to speak to her or the doctor enters the ward.

### TRAVEL NOTES AND QUERIES.

By our Travel Correspondent.

Convalescent Home in Ireland (E. M. B.).—If you will send me full particulars as to your terms I shall be pleased to recommend your house to any suitable applicants. We do not insert advertisements unless they are paid for.

The Pyrenees in Autumn (L. E. M.).—You were not answered before because early holiday-makers have to be considered first. October is a beautiful month in that district, but before deciding to go there, remember that the days will be short and the evenings long, so that you must be provided with indoor employments. Another difficulty is the expense. The journey each way, roughly speaking, will be about £3 10s. if you go third class, which is quite comfortable. I only know of two places where prices will come within the sum you mention. One is at St. Savin, in the Argelès Valley; address: Madame Jorly, Hôtel de la Vallée, St. Savin, Pres Argelès Haute Pyrenees. I know the place well, and found the hotel delightful and home-like, and the surroundings magnificent. You would be sufficiently near to visit Cauterets, and also Gavarnie, in a day's excursion. The other address is a private one, but I will give it to you should you fancy the place. It is 15 miles south of Oloron, is very primitive and quiet, and quite in the mountains.

### Novelties for Nurses.

(By our Shopping Correspondent.)

### POMADE MAX.

Pomade Max is a preparation of Ung. Cantharin with Hydrarg Co, thus blending a stimulant drug with a mercurial base, thereby removing largely cell infiltration and fibrous coagula in the skin and hair follicles which bring about atrophy of these structures and consequent loss of hair and loss of lustre. This will act not only as a preservative but as an adjuvant to renewal of robustness of hair when it has become thin and apt to split and degenerate.

### MENNEN'S TOILET POWDER.

Mennen's Toilet Powder has been in use for many years, and has obtained a well-merited and established reputation as a pure, soft, delicately-perfumed cosmetic powder. For summer rashes, all kinds of erythemas and skin irritations it is specially serviceable. For infants it is not only non-irritant but particularly soothing and healing, especially when the skin is delicate and liable to crack. Ladies will find it cooling and delicately fragrant when applied to the face, neck, or arms. The agents are Messrs. Lamont, Corliss and Co., 14 Queen Victoria Street, London, E.C.

### "REPELLO" CLINICAL THERMOMETER.

This thermometer saves a great deal of that irritative labour demanded by shaking down the column of mercury below the normal point each time the instrument has to be used. The "Repello" can be reset in an instant without shaking. In this way the number of breakages is reduced. The principle of action is very simple. The ordinary registering mercury bulb is at one end and at the other is another bulb, flat and circular, containing mercury, the two columns being separated by air. Heat causes the ordinary column to register the temperature; pressure of the top circular bulb causes the mercury in it to descend and repell the air and the registering mercury. It looks a delicate instrument, but is extremely easy, quick, and certain. It is a clever and useful invention, and should be a great boon to nurses as well as to practitioners. The inventor is Mr. G. H. Zeal, 82, Turnmill Street, London.

### COMBINED DOUCHE AND STERILISER.

We have received from this firm a douche can, as suggested by Dr. Claude St. Aubyn-Farrer, Physician Accoucheur and Lecturer on Midwifery, Royal Maternity Charity, London. It is a substantially made douche, fitted at the lower end with a brass nozzle for the attachment of a rubber tube, flattened behind, with a loop which enables it to be suspended at a suitable height when used for douching purposes. In order to fit it for use as a steriliser, a well made stand has been constructed, beneath which a spirit-lamp can be placed, and thus hot water can be readily obtained in cases of emergency, or in cases, common enough, in district or slum practice, where no utensils of any kind are at hand. This combined douche and steriliser obviates the difficulty so often experienced by midwives of being unable from one cause or another to obtain a sufficient supply of hot water. A hot douche can be obtained in a few minutes, and in cases where the surgeon's aid is required, the appliance can be used as a steriliser for his instruments, the can being large enough to hold forceps or other instruments which the surgeon may have to employ. Without the stand and lamp it forms an ordinary douche can, and the size is so convenient that it will readily go into the midwives' bag. It is supplied by the Medical Supply Association, 228-230 Gray's Inn Road, London, W.C.

### A Book and its Story.

### A MATTER OF OPINION.*

"The Coming of the Randolphs" is not an exciting story, but it is not without interest, and has all the good points associated with the name of the well-known author, who, as a writer of wholesome, if sometimes rather mild, novels, belongs to a school of fiction that is passing. The subject chosen is the well-worn one of a second marriage when each party has already made a previous venture. Mrs. Randolph, an early Victorian widow of a particularly trying type, who has begun "married life" some twenty years before, by running away with a man socially her inferior, has, in spite of her general mental incapacity, accompanied by a certain appealing prettiness, re-captured the heart of an old admirer, Colonel Underwood, a widower. He has one child, a daughter, Meriel, aged twenty-one. Mrs. Randolph has a family of seven children, ranging in age from three, to Hazel, the eldest, who acts the part of "little mother" to the turbulent party. Driven by the hopeless condition of her mother's affairs, Hazel has had to leave home and act as companion to a friend, an elderly lady. She is staying with her at Costabelle when the book begins.

"Miss Anderson was an old friend, and had taken Hazel as a companion during a winter abroad, partly in order to give the girl pleasure as well as to supply herself with a cheerful young companion. For Hazel was by nature gay as a lark, not easily tired or depressed, and very quick in devising comforts and kindnesses for other people. Miss Anderson declared that she was a treasure in the sick-room, and she only wondered what Hazel's mother did without her; to which Hazel would reply that there were so many of them that one did not count. Her figure was slight and graceful, but she was certainly not tall, a fact that Hazel Randolph lamented with all her heart. She was twenty-one, and had left off growing, so she would remain small to the end of the chapter. She was dark, with rough, curly hair, large dark eyes, and a little piquant brown face. Her features were not strictly correct, but her whole appearance was attractive, and the simple white frock and straw hat that she was wearing seemed, in its very simplicity, to enhance the wearer's charm." Hazel has come in from a stroll in the grounds of the Hotel at lovely Costabelle. Miss Anderson hands her some letters that are awaiting her. They are enclosed in a large envelope. This usually contained the home news as well as "bills sometimes, newspaper cuttings, or anything that Mrs. Randolph thought would interest her daughter. . . . It might seem most natural that she should begin to read her mother's letter first of all, but, as a matter of fact, she did nothing of the kind. She reserved it to the end, for although she loved her mother very dearly, a letter from Mrs. Randolph was not always an unmixed pleasure. It sometimes contained disquieting news and nearly always a good many complaints. Hazel liked to get a general idea of the condition of the family from her sisters' or brothers' letters first of all, so that she might have something to counter-balance the slightly depressing effects of her mother's correspondence." Hazel gathers from the unconnected tangle of indignant protest contained in her sister Kitty's letter that one thing at any rate is necessary, and that is her immediate return to the family. Kitty begs that she will come to rescue them from "this awful calamity. . . . It is not until she has read further that the nature of the "awful calamity" dawns on her startled eyes. The appeal of her sister to come home at once is repeated at the close of the letter: "We are all so miserable; we don't know what to do. Harry says he means to run away to sea, and Allen is dreadfully upset. He says he shall take a situation in a shop. . . . The only one of us who does not seem to mind is Jack, whom the Colonel has bribed with shillings and chocolates." The final appeal is insistent: "Dear Hazel, do come home as soon as you can, and tell Mamma that she must not do it." Then Hazel turns to her mother's letter. In it she finds abundant excuse for the step which would at one moment raise Mrs. Randolph from her present position of penury to one which promised all that was lacking in it—affluence, social position, someone to share with and relieve her of anxiety for the children's future. And yet, one wonders how a woman of so hopelessly weak a character could have attracted a man of Colonel Underwood's stamp. She is dominated by her children, and seems to have no will of her own. Hazel reads that her mother, being in serious pecuniary difficulties, turned in her distress to her old friend, Colonel Underwood, who had behaved with exemplary promptitude and saved the situation. He has crowned the occasion by an offer of marriage. His daughter required a chaperon, and he hoped to find in Mrs. Randolph the ideal one that he has been seeking, but, until now, had failed to find. Had Colonel Underwood read the letter that Mrs. Randolph sent to Hazel, he would, perhaps, have realised that he was mistaken. After many complaints of the behaviour of the children, whom she finds it impossible to control without Hazel's help, the letter proceeds:—

"If you had been at home I am not sure that the present difficulty would not have arisen, for I always longed to do the best for my children, and to sacrifice myself for them in every way. Colonel Underwood told me how attached he had always been to me. . . . I am sure we might all be perfectly happy if only the children would not set themselves against him and fly into such terrible rages at every little word I say." Hazel is as shocked at the thought of a second marriage for her mother as the rest of the family. But at this distance she is able to take a better balanced view. Hazel leaves for England at once, and learns from Miss Anderson before starting that Colonel Underwood has a daughter who is an acknowledged beauty and very devoted to her father. On her arrival at home a rather stormy scene has taken place between the mother and daughter. At its conclusion Hazel leaves her mother on the sofa and goes out.

"I wish I had never gone," said Hazel to herself, rather bitterly, as she closed the door softly behind her. When she meets her future father-in-law for the first time she is agreeably impressed; for Colonel Underwood was still a very handsome man, who carried himself with soldierly erectness. He had a quiet way of placing people at their ease with him, and he spoke to all women, even to little girls, Hazel noticed, with a kind of gentle deference, as though they belonged to a higher order of race than his own." In spite of Colonel Underwood's suggestion to adopt the whole family, Hazel is determined after the wedding, when she has seen her mother comfortably installed at Wood End, her new home, to return to London and keep house for the boys Allen and Harry, and to earn her own income. The marriage takes place, and Colonel and Mrs. Underwood go abroad at once, leaving Hazel to conduct the children to their new quarters and make the acquaintance of Meriel Underwood. Upon their arrival at Wood End, Hazel meets Captain Roden, whom she has known at Costabelle. His mother, Lady Roden, lives in the neighbourhood. Further acquaintance reveals that there is a near connection between her mother's and Lady Roden's family. The element of rivalry now enters into the story. Captain Roden has paid marked attention to Meriel Underwood for some time, and at Costabelle, he had spent much time in the company of pretty Miss Randolph also. The book runs on, with the interest well sustained, to a neatly worked-up conclusion.

* "The Coming of the Randolphs." By Adeline Sargent. (Methuen and Co. 6s.)

### Notes and Queries.

**REGULATIONS.**

**The Editor is always willing to answer in this column, without any fee, all reasonable questions, as soon as possible.**

**But the following rules must be carefully observed.**

**1. Every communication must be accompanied by the name and address of the writer.**

**2. The question must always bear upon nursing, directly or indirectly.**

**If an answer is required by letter a fee of half-a-crown must be enclosed with the note containing the inquiry.**

*Appointment Abroad.*

(236) Can you help me to find a post abroad or on the Continent. Can you give me address of nurses' club where I can consult a library of nursing literature.—*Nurse Ferne.*

You could advertise in the *Morning Post* and *New York Herald*, European edition. The Trained Nurses' Club, 12 Buckingham Street, Strand, has a complete library.

*Hospital Post.*

(237) What would be the best course to pursue to get a good hospital post?—*A. C.*

It would probably be best to secure a post as sister at a large hospital, and after a few years' experience seek the post of matron. You can watch advertisements or advertise yourself.

*Weir-Mitchell Treatment.*

(238) What is the name of Dr. Wood's Home or Institute in London?—*L. H.*

We do not give private addresses, but you can find Dr. Wood's address in the Medical Directory.

*Central Midwives Board.*

(239) Can I get the C.M.B. certificate without being a trained midwife, I have a certificate for monthly nursing?—*E. C.*

No. Get " How to Become a Midwife " from The Scientific Press, 28 Southampton Street, Strand, London, W.C.

*Colonial Nursing.*

(240) How can I obtain an appointment in the British Colonies or dependencies?—*Cicero.*

Write to the Colonial Nursing Association, Imperial Institute, S.W.

*Obesity and Gout.*

(241) Would very thinly cut and toasted bread be non-starchy and fit for a stout and gouty person?—*Dextrine.*

If the bread be cut exceedingly thin and well toasted it is permissible.

*Dried Milk.*

(242) Can you tell me where to obtain dried milk, and is it used for the same purpose as fresh milk?—*E. W.*

The West Surrey Dairy Company, Guildford, supply dried milk. It is used for the same purpose as fresh milk, the full amount of cream is not always present, and before it is given to an invalid a doctor should be consulted. It is a satisfactory and useful commodity for general use.

*Bandaging.*

(243) Where can I obtain lessons in bandaging?—*Merton.*

The St. John's Ambulance Society, Clerkenwell, holds classes at which bandaging is taught.

*Consumption Sanatoria.*

(244) Is there a free sanatorium for phthisical patients in England?—*C. S.*

Yes, there are County Sanatoria free to certain patients.

*Spinal Disease.*

(245) Can you tell me of a hospital which would receive a poor children's nurse suffering from a spinal complaint?—*Gant.*

This is a question best referred to a medical man, as a complete understanding of the case is necessary.

*Home for Aged Women.*

(246) Can you tell me of a home for an aged school mistress in London?—*School.*

The Woodside Home, Whetstone, N., £7 to £22 quarterly; the Westbourne Park Ladies' Home, 2 Shrewsbury Road, W.

**Handbooks for Nurses.**

| | Post Free. |
|---|---|
| " A Handbook for Nurses." (Dr. J. K. Watson) ... | 5s. 4d. |
| " Nurses' Pronouncing Dictionary of Medical Terms " | 2s. 0d. |
| " Art of Massage." (Creighton Hale.) ... ... ... | 6s. 0d. |
| " Surgical Bandaging and Dressings." (Johnson Smith.) ... ... ... ... ... ... ... | 2s. 0d. |
| " Hints on Tropical Fevers." (Sister Pollard.) ... | 1s. 8d. |

Of all booksellers or of The Scientific Press, Limited, 28 & 29 Southampton Street, Strand, London, W.C.

### For Reading to the Sick.

UNSEEN, YET NEAR.

Thou art near, yes, Lord, I feel it,
Thou art near where'er I move,
And though sense would fain conceal it,
Faith oft whispers it to love.

Then my soul, since God doth love thee,
Faint not, droop not, do not fear;
For though His Heaven is high above thee,
He Himself is ever near.

Near to watch thy wayward spirit,
Sometimes cold and careless grown;
But near too with grace and merit,
All thy Saviour's, thence thine own.

*J. S. Monsell.*

The soul which loves God for His own sake, gladly escapes from the business of the world to think of Him, recollects Him in little chinks and intervals of time, in which it is not occupied, takes occasion of all things to think of Him, is glad of hours of prayer to be with Him, glad to come to Him in His House and in His Sacraments, gathers itself together lest in the distractions of other things it lose Him.—*E. B. P.*

We live in the midst of revelations. We are continually receiving what we ordinarily call inspirations. There is hardly ever a complete silence in our souls. God is whispering to us well-nigh incessantly. Whenever the sounds of the world die out in the soul, or sink low, then we hear these whisperings of God. He is always whispering to us, only we do not always hear, because of the noise, hurry, and distraction which life causes as it rushes on.—*F. W. Faber.*

. . . In return for the love which brought the Son of Man down from Heaven, in return for the love which led Him to die for us upon the cross, we cannot give Him holy lives, for our lives are not holy; we cannot give Him pure souls, for our souls are not pure; but this one thing we can give, and this is what He asks, hearts that shall never cease, from this day forward till we reach the grave, to strive to be more like Him; to come nearer to Him; to root out from within us the sin that keeps us from Him."—*Bishop Temple.*

It is said that the fish of a certain river shine like gold so long as they are in their own waters, but take them thence and they become like common fish. Just so with afflictions; if we lose sight of God's Will they press upon us with all their inherent bitterness, but he who looks at them beneath the light of God's good pleasure sees them glowing, gilt, and precious.—*S. Francis de Sales.*

From strength to strength go on,
Wrestle and fight and pray;
Tread all the powers of darkness down,
And win the well-fought day.

That having all things done,
And all your conflicts past,
Ye may obtain, through Christ alone,
A crown of joy at last.

*Charles Wesley.*

## Figures and Tables

**Figure f1:**
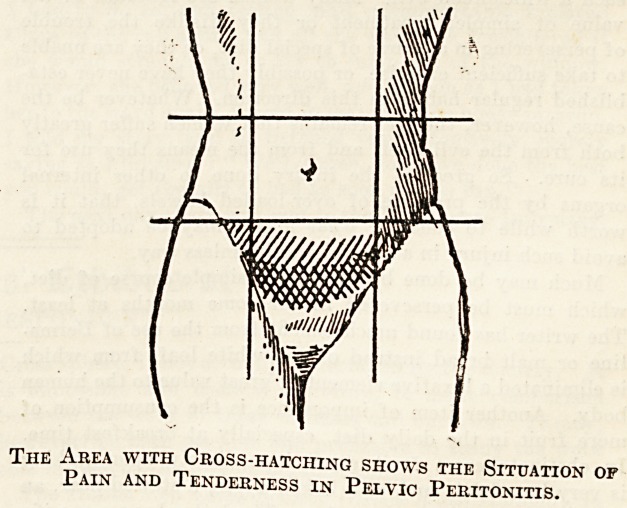


**Figure f2:**